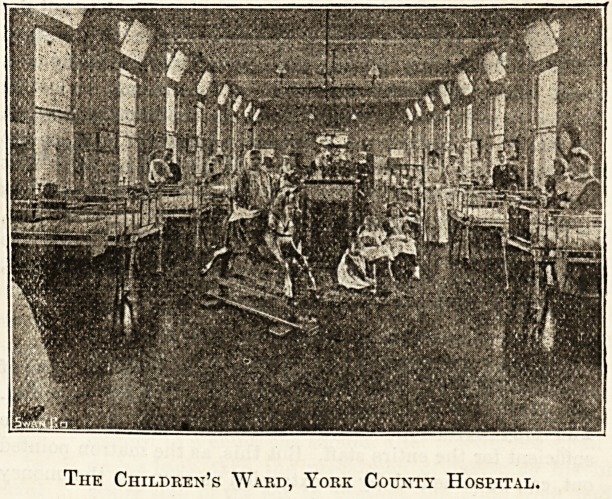# The Hospital. Nursing Section

**Published:** 1905-08-19

**Authors:** 


### The Hospital.

### Nursing Section.

Contributions for this Section of "The Hospital" should be addressed to the Editor, "The Hospital" Nursing Section, 28 & 29 Southampton Street, Strand, London, W.C.

No. 986.—Vol. XXXVIII. SATURDAY, AUGUST 19, 1905.

### Notes on News from the Nursing World.

### THE QUEEN AND THE NURSES.

It will be remembered that on the occasion of the recent Royal visit to Manchester, the members of the nursing staff at the Withington Workhouse were accommodated by the Guardians of the Chorlton Union on a stand erected for the purpose of viewing the procession. It was noticed that the Queen appeared to take a special interest in the nurses as she passed, and that she called the attention of the King to their presence. Dr. J. M. Rhodes, who is a member of the board, subsequently sent Her Majesty a series of photographs, showing the nurses at their duties in the hospital, and received in reply a letter from Miss Knollys, in which she stated that she was commanded by the Queen to thank Dr. Rhodes very much for the interesting photographs which he had been kind enough to send to Her Majesty for acceptance.

### QUEEN ALEXANDRA'S IMPERIAL MILITARY NURSING SERVICE.

We are officially informed that Miss M. Barton, Miss E. Close, and Miss M. M. McCreery have been appointed staff nurses in Queen Alexandra's Imperial Military Nursing Service; also that the following postings and transfers abroad have taken place:—Among sisters—Miss C. G. Stronach to Cairo, Miss M. Worthington to Alexandria, Miss A. A. Wilson to South Africa; and among staff nurses—Miss F. M. MacGregor to South Africa, Miss M. MacGregor to Cairo, Miss A. M. Pagan, to South Africa; also that Staff Nurse E. M. Bickerdike has been transferred from Woolwich to Alton. We are also informed that the following appointments as staff nurses have been confirmed:—Miss M. Clements, Miss S. N. Daly, Miss H. A. Hare, Miss D. M. C. Michell, Miss J. Murphy, Miss B. Rankin, Miss F. N. Roberts, Miss E. St. Quintin, Miss F. A. L. Smith, and Miss P. Steele.

### ROYAL VISIT TO RYDE HOSPITAL.

The King and Queen paid quite an unexpected visit to Ryde on Wednesday afternoon and inspected the Royal Isle of Wight County Hospital. The call was of an essentially private nature and was known to very few people. The Royal party came over from Cowes in three open carriages. The last contained the King and Queen, the Duke of Connaught, and H.R.H. Princess Henry of Battenberg, the president of the hospital. The King was received by the Rev. W. H. E. Welby (chairman of the committee) and Mrs. Welby, Mr. T. B. H. Cochrane (Deputy Governor of the Island), Mr. T. A. Buck, M.B., Lond. (the senior medical officer), and Miss Jones (the matron of the Convalescent Home), the matron of the hospital, Miss Antram, being away on holidays. The distinguished party visited all the wards and the new sanitary block. They spoke to many of the patients and took special interest in the children's ward, the memorial of Victoria's Jubilee, with which his Majesty expressed himself as particularly pleased. He took great notice of the bust of Queen Victoria by the late Onslow Ford, which surmounts the façade of the children's ward. Her Majesty said she had been very desirous of seeing this part of the building for some time. The King and Queen again expressed gratification with all they had seen, speaking of the bright, pleasing, and airy character of the wards. After taking tea the Royal visitors left the hospital. The late Dr. Davey, who was connected with the hospital for about forty years, and did much in securing the new children's ward, died immediately after its establishment was settled. He always cherished the hope that the King and Queen would visit the hospital.

### GIFT BY MISS NIGHTINGALE TO SCOTTISH NURSES.

The Queen's nurses in Scotland are indebted to Miss Florence Nightingale for a handsome gift of books which has been received from her at 29 Castle Terrace, Edinburgh, for the nurses' library. They are books of reference, and have been selected with a view to being of use to Queen's nurses throughout Scotland at their annual social meeting in Edinburgh on Jubilee Day. The present was accompanied by a communication, in Miss Nightingale's own handwriting, "To Scotch Nurses, with best wishes, from Florence Nightingale," and her thoughtfulness in sending the books will be not less valued than her generosity.

### THE QUESTION OF PREVIOUS TRAINING AT YORK HOSPITAL.

The Lady Superintendent of York County Hospital, in the course of an interview with our Special Commissioner, which appears in another column, states that the introduction of the system of three years' training has already had a beneficial effect. This and the completion of the Nurses' Home in 1903, marks that year as one of great importance in the history of the institution, which is now fully in line with the leading county hospitals. We are so often asked for the names of schools which do not object to candidates who have had previous training that some of our readers may be glad to learn that the matron of York Hospital is willing to receive as probationers nurses who have had children's training. As a matter of fact, her present staff includes two or three who have been trained in children's hospitals, and instead of the previous experience militating against their efficiency in general training, she appears to have found it rather an advantage.

### IMPORTANT PROSECUTION UNDER THE MIDWIVES ACT.

A case of considerable interest and importance was heard before Mr. De Grey at the South-Western Court on Saturday, the defendants being two midwives, who were charged, at the instance of the London County Council, with carrying on their business without obtaining a certificate from the Central Midwives Board. On behalf of the Council it was stated that this was the first prosecution of the kind under the Midwives Act. It was not disputed that both of the midwives held certificates from one of the London hospitals, but as it was clearly shown that the authority of the Board to practise had been refused, the magistrate was obliged to find that there had been a breach of the Act and required the defendants to pay the costs of the proceedings. He, however, advised them to appeal from the decision of the Board to the High Court of Justice, whereupon one of the midwives rejoined that she could not afford to do so. The Board are within their right in declining to give the reasons for their refusal to grant certificates, but it would have been more satisfactory to all concerned if, in this instance, they had waived it, and explained why two persons who have been in practice for many years have now been deprived of the privilege.

### VILLAGE NURSING IN NORTHAMPTONSHIRE.

A conference of members of Benefit Cottage Nursing Associations, working on Holt-Ockley lines, was recently held at Peterborough, the Countess of Ancaster in the chair. Several speeches were delivered, the principal being that of Lady Ancaster, who strongly urged the formation of new branches in the district. In the course of her address she referred in terms of praise to the highly-trained nurses working in Peterborough, but suggested that nurses of an inferior class are competent to deal with the ailments of the sick poor in agricultural localities. We recognise the fact that lack of sufficient funds may render it difficult to supply highly-trained nurses in some parts of the county of Northampton, as of other counties; but the village nurses who are employed under the auspices of the Benefit Cottage Nursing Associations ought only at best to be regarded as temporary substitutes for the qualified women whose services are as much needed and appreciated by agricultural labourers as by workmen in the towns.

### THE TRAINED NURSE ON SEA-GOING VESSELS.

The question of employing trained nurses on sea-going vessels has been raised again owing to the help rendered in the performance of an operation on board one of the ships of the Royal Mail Steam Packet Company by the trained nurse on board and by two passengers who happened to be nurses. The surgeon who performed the operation further remarks, that " without the willing and able assistance of the latter a successful result would probably not have been reached," by which we conclude day and night nursing was required. The importance of having a nurse on every ocean-going steamer has repeatedly been insisted upon by us. The need for fully-qualified women being employed, if they are to possess the confidence of the passengers and be really useful to the medical officer is again illustrated by this incident.

### EXONERATION OF AN ASYLUM ATTENDANT.

An inquest was held at Middlesex County Asylum on Monday on a patient whose death was alleged to be due to injuries received at the hands of one of the male attendants. The attendant implicated gave evidence, and stated that the deceased, who was an epileptic patient, and inclined to be excitable after his fits, struck him on the back of the head and put his arm around his throat while witness was going down a gallery with a coffee-pot in his hand. Finding that he was being strangled, witness tried to free himself from the grasp of deceased, and in the struggle they fell on the floor together. The patient, he added, was very violent, and witness had to seize him by the throat to save himself, but he had no idea that he was causing serious injury. The jury returned a verdict of " accidental death," and exonerated the attendant, whom they considered acted in self-defence.

### THE NEW MATRON OF MALTON HOSPITAL.

The importance of the duties of matron of the Cottage Hospital, Malton, is recognised by the authorities in the appointment of Miss Lilian Lloyd. She has enjoyed the advantage of varied experience. Trained at the Jessop Hospital for Women at Sheffield, and the General Infirmary, Bolton, she next spent some time under the Metropolitan Asylums Board at Park Fever Hospital. Afterwards she filled the responsible positions of night superintendent at Halifax Royal Infirmary, and at the Royal Hospital for Diseases of the Chest, London. She will have ample scope at the institution in the pleasant Yorkshire town for the exercise of her abilities.

### AUSTRALIAN NURSES IN LONDON.

An endeavour to dispel the loneliness of Australian nurses residing in London has been made by the Committee of Entertainment of the Victoria League, who recently communicated with His Excellency Sir Reginald Talbot, stating that it would gladly receive the names of those having personal introductions, and do all that could be done to welcome them and render their life in London comfortable. But the committee wish their visitors to realise that they can in no way be a channel to assist them to obtain professional engagements.

### COMPLIMENTS TO MATRONS.

At the annual meeting of the Royal Cornwall Infirmary, Truro, on Monday, the Chairman, Colonel Tremayne, said that Miss Rimington carried on the Convalescent Home economically and well, and the Hon. John Boscawen moved a vote of thanks to the matron of the infirmary, Miss Davis, who helped him in connection with the special fund, and collected for quite a dozen lockers. It was suggested that a portion of the balance of the special fund should be devoted to providing better recreation accommodation in the grounds for the nurses; but Mr. Boscawen rejoined that, whilst appreciating the nurses' work very highly, he did not see how anything could be taken from the balance for such a purpose. Subsequently the Chairman intimated that later on the committee might be able to do something for the nurses.

### The Nursing Outlook.

"From magnanimity, all fear above;
From nobler recompense, above applause,
Which owes to man's short outlook all its charm."

### DISTRICT NURSING AND ITS MAINTENANCE.

DISTRICT nursing means much to the poor, to the doctors and to the nurses themselves. The advent of the district nurse in a poor household in a large city has often given its members a glimpse of a new and better life. Disorder, discomfort, dirt, and insanitation are conditions which prevail still to a considerable extent in poorer households in great cities. The district nurse has to face and remove such conditions, to undertake to clean the rooms, and make them tidy and comfortable generally, in addition to her proper work in connection with the patient. Small wonder, then, if the poorer citizens regard the district nurse as a friend in need as they get to know her work and character, and recognise their value. The doctor finds the district nurse invaluable in many cases, because with her assistance he is able to treat them himself instead of allowing them to drift to the hospitals. As to the nurses themselves, we have always felt that a district nurse must be a woman of character and common sense, inspired by the motive of love, and possessing intuition and tact, with great power not only to make the best of things for the patient, but always to give satisfaction to the doctor. However much one may justly extol the life and mission of nurses in hospitals, it is essential that a due meed of special praise and thanks should be extended to the district nurses, who continue, year after year, their labour of love in the homes of the people.

The importance of district nursing to the whole community is becoming more and more generally recognised in these days. There can be no question that, where the poorer citizens are most crowded and congested in great cities, there an abundance of district nurses is essential to the well-being of the whole of the community. Indeed, it is not too much to say, that if it were possible, and it ought to be possible, to abolish the out-patient department of hospitals altogether, retaining the casualty department for minor accidents and urgent cases only, and substituting for the former a well-organised and complete system of district nursing, the benefits which would be conferred directly upon the people, and indirectly upon the medical profession and the community at large, would be incalculable. We are glad, therefore, that an influential deputation from the Queen Victoria Jubilee Institute for Nurses has approached the Hospital Sunday Fund, asking that the Institute may be given a grant from the Fund. The work of district nursing is cognate to the work of the hospitals, and it must tend to prevent many cases from finding their way to the out-patient departments which can be and ought to be treated in the homes of the people. At the meeting of the Council of the Sunday Fund last week a recommendation was brought forward to alter the laws of the Constitution so as to admit of a grant being made to the Queen Victoria Jubilee Nurses' Institute. After a discussion, it was, however, determined to refer the recommendation back to the Committee, in order that the condition of district nursing arrangements throughout London may be fully considered, and the effect and consequence of making grants to all district nursing associations in the metropolis may be fully ascertained. This decision marks an important advance in public opinion, and should result in hastening many necessary reforms in regard to out-patient treatment which have long called for redress.

District nursing in great cities, and especially in London, has been seriously handicapped in the past for want of adequate funds. In Liverpool the Hospital Sunday Fund authorities have recognised the importance of the work, and have made large grants in aid of district nursing. If the Metropolitan Hospital Sunday Fund is to deal with this matter it must take a wide and comprehensive view of all the questions involved. On a recent occasion we had the opportunity of seeing the work done in a congested portion of poorer London, where there is an active district nursing association. We found that the patients of the district nurse came through the local practitioners, the clergy, and ministers of all denominations, and the more intelligent and zealous workers among the poor. The organisation had existed for some years, with the result that the district nurses had obtained an intimate personal knowledge of the circumstances of the majority of the poorer residents, and their needs in regard to medical attendance and care. These facts prove that, assuming that London was broken up into centres, and that each centre had an adequate supply of district nurses to meet the requirements of the poorer residents, a reform in the present system of indiscriminate hospital relief could readily and promptly be put in force. It follows that the deputation to the Metropolitan Hospital Sunday Fund, coupled with the fact that the Distribution Committee of that Fund are at the present time taking steps to examine closely the present system of out-patient relief, may be attended with results, which will bring us nearer to adequate reforms in our present haphazard methods of medical relief, which must tend to strengthen the fibre and improve the morale of the people generally. Few if any people realised the possibility of such a magnificent result as the outcome of the establishment of the district nurse, which we owe so largely to that noble and zealous worker the late Mr. William Rathbone.

### Medical Electricity and Light Treatment.

By Kate Neale, Sister-in-Charge of the Actino-Therapeutic Department, Guy's Hospital.

### V.—STATIC ELECTRICITY.

You will remember that I have mentioned two kinds of electricity, static and current. The application of the latter you have already learnt in the preceding pages, and it is to treatment by the former that the present chapter will be devoted.

The static electricity produced by rubbing silk and glass together is not sufficient to be of any medical use, and for this purpose we have to employ a special apparatus or machine from which a much greater amount of electricity can be obtained. There have been many forms of machines devised in the past 150 years or so, but the one almost universally adopted for medical work at the present day is that known as "Wimshurst's Machine."

### Wimshurst Machine.

This is shown in fig. 7. It consists of a number of circular glass plates (usually eight) which are

7. Wimshurt Machine.

made to revolve, some in one direction some in another, either by a handle, or preferably by a special electric motor. They are enclosed in a glass case which should be proof to dust and damp. On the front of this case are the two terminal poles, A and B, one being the anode and the other the kathode. Each consists of a metal rod ending at one extremity in a metal knob, and at the other in a vulcanite insulating handle. They are known as the **conductors**, and by means of the handles their knobs can be separated or brought together.

As soon as the machine starts moving, positive electricity collects at one conductor, negative at the other, and, if the knobs are sufficiently close, bright sparks will leap across between them. The simplest method of treatment is to charge the patient with electricity (positive or negative as the case may be) by putting him in communication with one conductor while the other is connected to the ground —"earthed" it is called—by a long metal chain to allow its charge to escape. In addition to the machine itself certain other appliances are needed such as an insulating stool and electrodes.

### Insulating Stool.

If you connected one conductor to the patient, as just mentioned, but at the same time allowed him to stand on the floor, his charge would at once escape to the earth. To avoid this he must be placed on a special stool whereby he is "insulated" and his charge retained. The platform of the stool is made of wood, all corners being rounded off, and rests on four strong glass legs. Glass, we have seen, is a non-conductor, and the legs will therefore prevent any escape of charge. The stool should be of such a height that when not in use it can be pushed out of the way underneath the Wimshurst machine. Fixed to the platform there is often a brass plate which can be connected by a metal chain to one of the conductors, but a better plan for joining patient and conductor is to use a metal rod or tube about three feet long, bent at one end like a shepherd's crook. This crook is hooked round the conductor, and the free end is grasped by the patient.

### Electrodes.

Statical treatment is applied by some form of electrode, of which there are five varieties, all except the last being provided with a vulcanite insulating handle:—1, point electrode; 2, fork electrode; 3, ball electrode; 4, roller; 5, tassel.

8. Static Electrode.

No matter what form of electrode is used, it must always be earthed by the agency of a long metal chain, one end of which is fastened to the eye of the electrode and the other to a hook specially provided on a leg of the machine case. A **point electrode** (fig. 8, A) is shaped rather like a big lead

MEDICAL ELECTRICITY AND LIGHT TREATMENT—*Continued.*

pencil, and is made up of a pointed metal half and a vulcanite insulating handle. In some cases a **fork electrode** with many points is required. Such a one is seen at B, and ends in a metal disc set with thirty to forty spikes. The **ball electrode** C, differs from the preceding in that it has a terminal ball about the size of a small orange. The **roller** D works in the same way as a squeegee, and is run rapidly to and fro over the surface of the body. The **tassel** is specially used in applications to the head. It is shown in fig. 7, hanging from the top of the machine by a metal rod, which is connected to the case and so to the earth.

### Methods of Treatment.

There are many methods of treatment, but all depend on the same underlying principle. In every case the patient, insulated on the stool, is connected by means of the crook to one conductor while the other is joined by a long chain to the hook on the leg of the machine. The patient becomes for the nonce, a part of the machine and is charged with the same electricity as is the conductor to which he is attached. The charge in the other conductor runs away to earth as soon as it is formed. Put your finger within a few inches of the patient, and some of his charge will flash as a long bright spark from him to you. Sometimes he has to be charged positively and sometimes negatively, and you must therefore be able to identify anode and kathode. This is the more necessary because the polarity of the machine is not constant, and what at one time is the positive conductor may become the negative when next the machine is used.

### To Identify the Conductors.

Set the machine in motion and present the point electrode, earthed in the usual way, to the knob of one conductor. Hold it at first some inches off, and approach it gradually nearer and nearer. You will see, flickering around the point of the electrode, a halo of bluish light, which, in the case of the positive conductor will grow brighter and larger, until at a distance of one to two inches short crackling sparks will spring across the gap. If, however, it is to the negative conductor you are directing the electrode, large and brilliant sparks will appear while the interval is still of many inches.

The differences in the various methods of treatment represent merely differences in the manner of charging and discharging the patient. These methods are four in number, though each is again subdivided.

I. Simple Charge.
  (1) Positive; (2) Negative.
II. Charge and Discharge.
  (1) Positive; (2) Negative.
III. Static Breeze.
  (1) Positive; (2) Negative; (3) Head Breeze.
IV. Static Sparks.
  (1) Positive; (2) Negative; (3) Roller.

There are many details which have to be observed, whatever form of treatment is ordered. These I shall consider before noticing the points peculiar to each of the four groups.

In the first place, a Wimshurst machine often shows an annoying disinclination to work if the weather be cold or damp. For this reason its case, as I have said, must be damp-proof, and the room in which the machine is established should be provided with a fire or heat-radiator. But even with these aids you must be prepared to meet with disappointing results on wet or muggy days. With rare exceptions treatment is always given with the patient fully dressed, and there is no occasion to remove any garment except the hat and sometimes the shoes. Once the patient is charged on the insulating stool you must be careful not to approach too near lest painful sparks may be drawn from him. Even if your propinquity is not close enough to allow this, there may be a transference of electricity from you, which he will feel as a distinct breeze blowing towards him. Keep well away from him then, and allow none other to approach; remove all articles of furniture from his neighbourhood; and, lastly, take care that the stool is a good two feet from the machine-case or walls of the room. The usual duration of treatment is from ten to fifteen minutes, though in the more stimulating forms—such as sparking or roller-friction—the time ordered may be shorter. At the conclusion of the treatment bring the machine to rest, and discharge the patient by placing one foot on the stool, when his electricity will gently leak away to earth through you without either feeling a shock.

*(To be continued.)*

### The Nurses' Clinic.

### NASAL FEEDING.—BY A SISTER.

Nasal-feeding is frequently ordered where there is great difficulty in taking food by the mouth, as in cases of alveolar or parotid abscess, or where it is almost an impossibility, as in tetanus. It is also resorted to when the patient is hysterical, delirous, or demented, and keeps his mouth firmly closed whenever an attempt is made to feed him or to administer medicine.

Nasal feeding is far more often used for children than for adults. Some doctors like their tracheotomy cases to be fed in this way; occasionally nasal feeding has been successful in cases of persistent vomiting after food, and it is very frequently prescribed in broncho-pneumonia, or, indeed, in any ailment where the child absolutely refuses to swallow, and where the excitement and exhaustion consequent on forcibly feeding it by the mouth would be harmful. Children, as a rule, make little or no resistance to nasal feeding after the first time; indeed, one baby of my acquaintance has been known to laugh and smile at the nurses during the process. When an adult has to be fed through a nasal tube the nurse must have assistance; in fact, it is always advisable, but, if she cannot get help, and is obliged to give a nasal feed single-handed to a child, she should, after placing her small patient on his back, take the precaution of fastening his hands to either side of the cot to prevent the child from pulling the tube away. If it is a baby that she is to feed, the best plan is to roll it in a blanket, with its arms to its sides, securing the blanket with safety pins.

For nasal feeding the nurse will require the following articles :—

1. A new soft rubber catheter, varying in size according to the age of the patient, whether baby, child, or adult, the smallest size made will be needed for a baby.
2. A glass funnel of a size to fit into the catheter.
3. A bowl of warm boric lotion.
4. Two receivers, one to hold the funnel and catheter when used, and one for use in case the patient should vomit.
5. Some sort of lubricant. Glycerine is apt to irritate the mucous membrane, vaseline or olive oil are better.
6. A towel, to place under the patient's chin.
7. And last, but not least, the food and any medicine or stimulant ordered to be given with it or after it. The food should be warm, and it is best to have it in a glass measure, it is easier then to be certain of the exact amount taken.

The catheter and funnel, having been boiled, should be connected and put into the boric lotion. The patient should lie on his back, with his head slightly raised on a pillow and the nurse assisting, must stand at his right side, ready to steady his head and hold his hands should he attempt to clutch the tube out of his nose. The nurse who is to give the food must be sure that she has everything she requires ready by the patient's bedside before she begins operations. She must lubricate the end of the catheter and pour some of the lotion through both funnel and catheter, to be sure she has not blocked the eye in so doing. Then, holding the catheter in the right hand as she would a pen, she must pass it firmly and gently through the nose into the throat, directing it backwards along the nasal septum. The distance between the front teeth and the beginning of the œsophagus or gullet is about six inches, and the nasal passage from the nostril through the posterior nares into the pharynx, is about four and a half in the adult. So that if five inches of the catheter be passed into the nose, the eye should have arrived in the pharynx. The nurse should bear in mind the anatomy and relation of the parts concerned when using a nasal tube. She must remember that, instead of the tube passing over the epiglottis, as it would do if passed through the mouth, it passes behind it when on its way from the nose, and, if passed too far, it may go under the epiglottis into the trachea or windpipe instead of into the epiglottis. This very rarely occurs, but the possibility of such an accident must be borne in mind. Should it happen, a violent fit of coughing will result. The catheter must, of course, be instantly withdrawn, being passed again when the coughing has subsided and the patient has quieted down. Another thing that sometimes happens is this: the tube, instead of going into the throat, curls forward into the mouth and appears between the teeth. This can generally be avoided by closing the patient's mouth and pressing the jaw upwards, with the finger placed far back under the chin. Should there be resistance to the passage of the catheter, or, in the case of a child, a fit of crying, it is advisable to wait with the catheter in the nostril and pass it as the patient gives out rather than takes in his breath—that is to say, during expiration rather than inspiration, he is then less likely to draw it into his mouth. When the catheter has been satisfactorily passed, about a teaspoonful of the food should be poured into the funnel, and if there is no coughing the remainder can be given and the catheter be rapidly withdrawn when it is finished. Sometimes the food remains stationary in the funnel and will not pass down the tube – the eye of the catheter may be pressing against part of the throat, and the fluid prevented from flowing out. Withdrawing the catheter a little, or passing it a very little further, will usually overcome this. But if the funnel still remains full it is probable that the eye of the catheter has become blocked, in which case the food must be emptied back into the glass measure and the catheter must be withdrawn and cleaned and then be passed again. When nasal feeding has to be repeated it is well to pass the tube through either nostril alternately, so as to avoid irritating the mucous membrane as much as possible. Even with every care, if nasal feeding is long continued some inflammation and swelling may result, and it is often necessary to change the catheter for a smaller one, as the passage becomes narrower. Care should be taken to gently swab out the nostril with boric lotion and to use a little vaseline, both inside and out, after each feed, and, of course, to sterilise the catheter and funnel each time they are used, leaving them ready in the lotion for the next occasion, covered with a folded towel.

### The Nurses of York County Hospital.

### INTERVIEW WITH THE LADY SUPERINTENDENT.—BY OUR COMMISSIONER.

It is not very long ago since The Hospital had to tell the committee of the York County Hospital that in the arrangement of the nursing branch, the hospital was unworthy of a great county institution, and that no time should be lost in taking steps to erect a nurses' home on the latest principles. As Mr. W. W. Hargrove, a well-known citizen of York, in the history of the city which he is now publishing, says, that slur was removed in March 1903 when the new home was opened. On the occasion of my visit to York the other day, I had the opportunity of inspecting this building under the auspices of the lady superintendent, Miss A. M. Edwards, who also showed me over the hospital.

### The Nurses' Home.

It is entirely on modern lines and is wanting in none of the provisions which are now rightly considered essential. The only defect is that the accommodation is not quite sufficient for the entire staff. But this, as the matron pointed out, can be remedied by an extension as soon as the money available for the purpose is forthcoming.

" At present," she said, " there are separate bedrooms for twenty nurses, as well as a general sitting-room, several bathrooms, and other necessary accommodation."

" The sitting-room, which is commodious and tastefully furnished, contains, I see, a small library and a piano."

" The piano has just been given to us, and it is a very good instrument."

" You seem," I said, as we looked into one of the bedrooms, " to have aimed at securing the maximum of comfort without indulging in luxuries ? "

" That was what the committee in planning and furnishing the home set themselves to accomplish. Each bedroom, you notice, has a black enamel spring bedstead, an oak dressing-table and washstand, a bedside table, a couple of chairs, and a hanging wardrobe, the last being a fixture."

" And also a fireplace ? "

" Yes, I think that a fireplace, both for ventilation and in case of slight indisposition, is very requisite. The whole of the top floor is given up to the night nurses, and the building throughout, like a portion of the hospital, is lighted by the electric light."

" Have you a room of your own in the home ? "

" Yes, I have rooms in both the home and the hospital, but I spend most of my time in the latter."

" The number of nurses is considerably more than 20 ? "

" There are 27 probationers and eight sisters. With

### THE NURSES OF YORK COUNTY HOSPITAL—*Continued.*

regard to other sleeping accommodation, there are ten very superior cubicles in the old block, quite away from the wards, where some of the junior probationers still sleep.

"Meals, I presume, are served in the hospital."

"In the dining-room in the main block. The nurses may have tea in the home if they like to get it for themselves.

A GROUP OF NURSES, YORK COUNTY HOSPITAL.

But no meals are served here, and no food is allowed in the bedrooms, except breakfast in bed by my special permission."

Finishing at this point our inspection of the home, we proceeded to the exceedingly bright and well-equipped children's ward, which was only added to the hospital in 1898, spent a little time in some ordinary wards, and in the handsome operating theatre, which was remodelled last year. I also noticed that the nurses have a shady lawn of their own, that there are well kept lawns at the back of the hospital, and that a number of the patients were enjoying the air on the outside balconies.

### THE TRAINING.

"The three years' training is quite a recent innovation?" I said inquiringly, when we had returned to the matron's pleasant room in the hospital.

"Originally the training was only for one year. It was afterwards increased to two, and during the matronship of Miss Forrest was extended to three years. As a matter of fact, when the two-year system was in force, many of the nurses who came for two stayed for three years. Another recent alteration is that the preliminary training has been increased from one month to two. In making this change, I was influenced by the conviction that a month is not a sufficient time in which to test the fitness of a candidate for the part of probationer. Of course the two months are included in the three years' training. Before they sign on they have to pass a medical examination."

"Is there any special feature in the theoretical training?"

"I think not. There are three courses of lectures during the year from the medical staff, and at each successful examination a certificate is given, the certificate of training being given at the end of the third year."

### THE QUESTION OF PREVIOUS EXPERIENCE.

"What is the age of admission?"

"It was 21, but it has lately been raised to 23, and we do not admit probationers after 30. A great many matrons of general hospitals will not accept a candidate who has had children's training, but I do not object to it. In fact I have now two or three nurses who have had children's training, and it has not militated against their efficiency in general nursing."

"Moreover, the nursing of children here is very considerable?"

"The children's ward has 20 beds as against 13 in the old ward. There is a sister in charge, and always three nurses on duty in the day, with one at night or a second if the work is heavy. Another feature here is that we are permitted to take eight typhoid cases at a time. These being dispersed through the medical wards. As there is no medical school the nurses have the joy of doing work, instead of the less welcome task of merely clearing up."

### VARIETY OF WORK.

"Then there is the ophthalmic ward?"

"Yes, and it has a separate theatre attached. There are 13 beds, and a sister and one nurse are on duty. I endeavour to pass each nurse through every ward, so that she may obtain as great a variety of work as possible. With regard to the operating theatre, there is a sister in charge and a nurse to assist. Each nurse has three months' experience in the theatre. We have a very large number of casualty cases, and the out-patients' department is an important branch."

### PAYING AND NON-PAYING PROBATIONERS.

"I think that you have both paying and non-paying probationers?"

"Paying probationers used to pay a premium of £20; it

THE CHILDREN'S WARD, YORK COUNTY HOSPITAL.

has been reduced to £10. I had to refuse suitable women because they could not afford to pay £20. The paying probationers, however, have a salary of £9 the second and

£11 the third year. As the non-paying probationer only gets £5 for the second and £5 the third year, you see that there is practically no difference. I get an ample number of applications for vacancies, more, indeed, than I can consider."

"What uniform do you provide?"

"Both indoor and outdoor, but the wearing of outdoor uniform is optional. My nurses appreciate uniform for outdoor use. It means the saving of time when they are only off duty for a short while, and economy in dress."

### Long Holidays.

"With respect to the hours of duty," continued the lady superintendent, "our rules have just been revised, and are up to date. The probationers breakfast at 6.30, are on duty at 7, and off duty at 8.30 p.m. They are off duty one day in each week from 3 p.m. until 9.30 p.m., and, as far as I can manage it, they have one and a-half hour's recreation each day. The probationers are supposed to have three weeks' holiday the first year, but practically every nurse, as well as every sister, gets a month's holiday. I think that they really need it."

"How many nurses do you assign to the double wards?"

"A sister, a senior nurse, and two junior probationers. The large surgical wards contain 26 beds, and the large medical 24 beds. In the single wards, with 13 beds, a sister and a probationer are always on duty. At night the hospital is in charge of the night sister, and there is a nurse on duty in each ward. In a very bad case I put on a second nurse. Night nurses come off duty at 8 a.m., and have to be in their bedrooms at 11.30. They get two and a half hours off duty each day, and a night off once a month. Every sister and nurse has four hours off duty on Sunday, and we make the work as light as possible in order that all the nurses may have a little extra rest."

"When does a probationer first go on duty at night?"

"At the end of nine months. I am sure that it is a mistake to give a nurse night duty until she has got accustomed to be on the alert. I am very particular in requiring nurses on night duty to spend at least eight hours in bed daily."

### Dress and Recreation.

"In your rules I observe that nurses are reminded that high heeled boots and shoes and fringes to the hair are unsuitable for a hospital."

"Yes, that and some of the other rules may seem trivial, but they are most important from a nursing point of view. With respect to dress generally, I like dresses to be made quite plainly in bodice and skirt, length just off the ground, and sleeves neither high nor full; aprons to be made with bib and straps, the apron to come within two inches of the bottom of the dress, and the bib to be long enough to meet the collar. No brooches, rings, or ornaments of any kind can be worn. As to recreation, most of the staff have bicycles, and this year a room was fitted up for the housing of the machines. I believe in nurses having as much exercise as possible. There are picnics for them during the summer, up the river and elsewhere."

"Were most of your sisters trained here?"

"Only one was trained elsewhere and she has been here more than three years. But our nurses have no difficulty in getting good appointments in other institutions. They are now eligible for appointment to Queen Alexandra's Imperial Military Nursing Service, and I think I may say that York County Hospital stands very well as a training school."

### An English Nurse in a German Kurhaus.

We started on a 40-horse power Daimler motor car, and covered the first six miles of our long journey (North Staffordshire to Bad Kissingen) in about ten minutes, three of which were spent in waiting for the gates of a level-crossing to be opened for us. It was both pleasant and exhilarating to move so rapidly through the air for a short distance, but I felt glad both for my patient's sake and my own that we were not going to travel far in such an exciting manner. We then joined a train for London which should have reached town in time for us to get a comfortable meal at Liverpool Street, but some mischance kept us for an hour outside Euston, so it was all we could do to catch the boat train for Harwich. By the time we reached the latter place rain was falling heavily, it was dark and there was a great scarcity of porters; we had seven pieces of hand-luggage, and it was with difficulty that I got it all, and my patient and myself conveyed on to the steamer only just as the last bell was ringing. That was the beginning of the most wretchedly uncomfortable night that I ever passed. The boat was crowded with passengers, and for some little time after we got on board all around us was a scene of wild confusion. All were eager to get into bed before they felt ill; some had engaged berths, others had not been so prudent; no one knew where his or her berth was to be found. I had reserved a double berthed cabin, but it was ten minutes or more before I could get my weary little lady and all her various belongings safely established there. I put her to bed, but she felt too ill and wretched to allow me to lie down on my berth, even though I too suffered badly from mal-de-mer. The stewardess was of no use whatever. She settled herself comfortably down on a sofa in the saloon and simply ignored the bells and the piteous cries which resounded constantly all around us through the night. It was not a particularly rough night, only "half a gale" of wind and heavy rain, but every opening which might have admitted air was closed, and the movement of the boat and the atmosphere were indescribably horrid.

### Arrival in Holland.

It was still raining when we landed at the Hook, but the express train by which we were to travel through Rotterdam to Cologne, was waiting quite near to the landing-stage. The douane was soon passed through and we secured good seats; a friend in need showed me where I could get hot water for the foot warmer, and I soon had my very unhappy patient so comfortably settled that she cheered up and began to enjoy the picturesque Dutch scenery through which we were passing; so that I was able to think about making myself more comfortable and "fit to be seen." I found a good supply of water and a clean basin in the lavatory attached to the corridor carriage, also an "Automat" out of which, by means of a 10 Pfg. piece I got a small packet containing soap, an antiseptic hand-towel, toilet paper, etc. Such an excellent arrangement! I was soon clean and tidy, and felt so refreshed as to be quite ready for breakfast, which was shortly served in the Speise-Wagen. How much our English railway companies might learn from those on the Continent! Instead of having to pay the fixed price of 2s. 6d. for an unnecessary variety of heavy articles of food (bacon, eggs, cutlets, fish, etc., etc.), we were allowed to order *à la carte*, and were charged only for what we actually ate.

### Up the Rhine.

Cologne was reached about noon. There we stayed for the night. In the afternoon we did a little shopping, crossed the

### AN ENGLISH NURSE IN A GERMAN KURHAUS—*Continued.*

Bridge of Boats, and went for a few minutes into the glorious cathedral. For the sake of quiet we chose two small rooms at the top of the great Dom Hotel. There we supped early, had a good night, and breakfasted in the early morning. At 8.30 we were in our places on the luxurious steamer which was to take us up the Rhine as far as Mayence. This day on the Rhine I had arranged for as being less fatiguing for my nervous patient than continuous railway travelling. Again it rained, but we were able to sit on deck all day under the awning, only going into the saloon for our meals, and undoubtedly the low clouds and mistiness rather added to the picturesqueness of the Rhine scenery; the rocks and mountains appeared to be higher and grander than usual, the castles more romantic, the vineyards less monotonous. We reached Mayence just as it got dark, about 9 P.M., selected pleasant rooms in the Holländische Hof, and soon got a fire lit in the big stove in the corner, around which I dried rugs, shoes, cushions, etc., whilst we had supper and prepared for bed. A stroll in the interesting old town the next morning; a little shopping (picture postcards, milk for the tea-basket, etc.), and at noon we started again; this time by rail for the last stage of our journey. Again, a comfortable carriage (second class, but quite as good as our first); this time our companions were Germans, but they were most kind and friendly. A stout, elderly man gave up his corner seat to my patient, and was attentive to her comfort all the afternoon. Another man and his wife, also going to Kissingen, gave us much interesting information and also asked many questions And all three were intensely interested when I presently proceeded to unpack the tea-basket; they agreed that the operation was "sehr gefährlich" (very dangerous), but still they kindly persisted in helping at it. One undertook to hold the bottle of milk, the second watched the spirit-lamp, and the third tasted the Plasmon biscuits.

### Kissingen.

About 6 P.M. we reached Kissingen, a terminus to which we had for the last hour been gradually rising through a well-wooded mountainous district dotted over with little villages, that were just groups of red-roofed houses, generally clustering round a church, and surrounded by orchards and vineyards. The wild flowers were very beautiful, most of them like our English ones, only growing more luxuriantly than they do with us; great masses of scarlet poppies, blue corn-flowers, and white moon-daisies; but there were also others, which were strange to us. On the platform at Kissingen I was relieved to find my patients' heavy luggage, which I had registered through from London, awaiting our arrival, and very quickly we got it and ourselves into an open carriage, in which to drive to the Kurhaus.

### Our Quarters.

Down a long avenue of trees, through the town, over the river, past the large Casino, with its public gardens, wells, band, and tennis-courts, then up hill to our own Kurhaus. We were received by a pleasant little lady, who essayed to greet us in English, but looked much relieved to hear that that was not necessary. She explained that, because the house was so full, I should have to sleep in a neighbouring villa; so my bag was left in the hall, and my patient and I were taken up to her room, and told that the evening meal would be ready in about ten minutes. This was a cheerful little room, but with the usual Continental peculiarities; a wooden bedstead in one corner with great square down pillows and a huge feather bed (meant to be slept under), no fire-place, no toilet table, looking-glass on the wall over the wash-stand, which stood so close to the window as to be in full view of the "Kurgäste" as they lay in the garden on their couches under the trees; a large sofa in the darkest corner of the room, a white cloth spread cornerwise on the writing table, a polished floor with two bright rugs, no pictures or ornaments, but a set of printed rules on the wall. All exquisitely clean, and the air so pure and refreshing that I felt as though I would like to be starting a two months' cure myself. But my patient was tired and on the verge of a nervous breakdown. I made haste to find her fresh clothes and to help her to change into them; only being able myself just to wash my hands and face before the gong sounded. We easily found our way down to the dining-rooms—three clean, airy rooms opening one into another, evidently furnished only for the purpose of dining in, polished floors, long narrow tables, cane-bottomed chairs, black-handled knives and forks, teaspoons, and a knife-rest for each person. In Germany the same knife and fork serves for the whole meal.

### The Diet.

About forty persons sat down; some looked rather ailing, but most appeared to be in perfect health. All were cheery and particularly polite, for on entering and leaving the room each one always bowed and saluted his neighbours. Every person's diet is ordered by the doctor himself, and the diets are served in neat little white china dishes, with the room number of the person for whom it is meant at the side. Not only are you required to abstain from eating anything which is not ordered for you personally, but you are also required not to leave uneaten any of your own portion. I do not mean that the food is absolutely forced down one's throat, but the doctor walks to and fro through the dining-rooms whilst the meals are going on, and he looks very grave if he sees the big square dishes with any of the rice left in them, and I was told that he declines to keep any guest in his house who does not at least try to carry out his instructions. No pepper is allowed and no mustard. Salt must be used sparingly. The food was very good, well cooked, and well served; but it seemed to me that the sameness of it would become very monotonous to anyone who stayed long—two or three months in the house. Even the bread was varied only according to the patients' personal needs. No one could choose each day which he would take. Some had the dainty little "brödchen" which are met with everywhere on the Continent, others "schwarzes" bread (I believe made from rye), others honey bread, others delicious toast made from a very short and rather sweet loaf, others a sort of rusks, also very good. Apparently only those who were afflicted with excessive corpulence got beef and mutton; those suffering from some form of gastric or bowel trouble (and by far the greater proportion of the guests belonged to this latter class) were served with chicken (cooked in oil), veal cutlets, pieces of liver, or fish, followed by rice cooked in milk. Of the latter, deep square dishes full, portions of about 1 lb., were rapidly and easily disposed of by those who had been some weeks in the house and got accustomed to the diet, but the new-comers found considerable difficulty in eating it. It seems that the German doctors consider this form of milk food to be more nourishing and more easily digested than simple milk. Breakfast is served separately—in bed for some, in the garden for others, so I did not see what most people got; probably the same as was given to us—namely, eggs, weak tea or coffee, and toast, with a little butter. The midday meal is like the evening one. Afternoon tea is allowed to some patients, but it is served without any leaves in the pot—weak China tea.

### Rest and Baths.

But the diet, although considered to be of great importance, is not by any means the chief part of the treatment. The doctor believes in rest for most of his patients—not the fat ones. They have to rise at 5 A.M., walk before breakfast and after breakfast, again in the afternoon, and again in the evening. Rest in bed after the mid-day meal for two hours, and rest in the garden on comfortable couches (just like beds) for the greater part of the morning and evening: twice a day for some, only once for others, a short walk—15 to 20 minutes—is ordered. No exciting work or talk is allowed, just a light book over which to fall asleep. Then there are baths, rubbings with alcohol, massage and electrical treatment, compresses and injections, ordered for each patient according to his or her necessities. There are no nurses in the house; the treatment is carried out, under the doctor's supervision, by an assistant doctor, a masseur and a masseuse, and a "bath woman," aided by chambermaids. Two young ladies act as secretaries—one for the house affairs, the other for the doctor himself, with his copious notes, which are most carefully made about each patient, both as to condition on arrival and the progress made. And so we come to that part of the whole business which impressed me most strongly—namely, the extraordinary care which the doctor takes in the examination of his patients.

### The Examination.

I have never seen anything like so thorough an investigation in England or Scotland, either in or out of hospital. Not all at once. In the two days before I left the Kurhaus my patient was seen four times, and even then the doctor did not consider that he knew enough of her case to entitle him to order her treatment. A large consulting-room, divided by a screen, on one side of which sat the secretary ready to write from the doctor's dictation; on the other side a couch for the patient to lie upon, everything close at hand which the doctor conld possibly need. Height and weight were taken; skin, eyes, ears, nostrils, mouth, throat, tongue, all examined separately and noted; general appearance commented upon, all the limbs and the principal muscles felt; heart, lungs, stomach, liver, spleen, bowels, all mapped out on the body with chalk and then measured; heart, lungs, and pulse thoroughly examined, the saliva tested, the contents of the stomach (reached by means of the stomach-pump) chemically examined. And then the questions! Not only must her own history be found out, but, so far as possible, that also of her father and mother, her brothers, sisters, and such clever questions were asked as to her symptoms; I mean by that, questions put in such a way that the answers must necessarily give a real insight into her condition. He gently checked her when she occasionally ventured to volunteer information, and indeed his method was undoubtedly the best possible.

### The Instructions to the Patient.

After the first three or four days the doctor makes a diagnosis, decides upon a course of treatment and gives the patient a paper of instructions; instructions for every minute of the day and the patients are required not to alter any minutest detail without permission from him. He can be seen in the consulting-room at stated times, or if any one is ill in bed he will visit the patient there two or three times a day. Except the eating of the rice and the stomach-pump process, I think that it would be a delightful experience to undergo a two months' cure in the M—— Kurhaus. To spend many hours each day lying on an easy couch in the shade of lovely trees, roses, carnations, heliotrope and other sweet scented flowers blooming freely close by, pleasant companions around one, occasional strains of music rising from the public gardens; a kindly sympathetic doctor to direct all one's actions; could there be a happier existence for a while for a tired-out private nurse?

### The Registration of Nurses.

### A PROTEST AGAINST YELLOW JOURNALISM.

In dealing with the recommendation of the Select Committee on Registration to set up a General Nursing Council in our issue of the 5th inst. we wrote: "Providing all the interests involved are adequately represented on the General Nursing Council this recommendation is likely to gain general acceptance. It will be seen that the Committee, whilst recognising the impracticability of the schemes of registration so far submitted to Parliament, are nevertheless of opinion that it is desirable that a register of nurses should be kept by a representative nursing council appointed by the State, and with this in view it is desirable to bring about with all possible despatch the coordination of the various training schools throughout the country." We thus recommended the closing of the nursing ranks, and the union of all parties which have the best interests of nurses at heart. The words bear no other interpretation.

In the *British Journal of Nursing* of the 12th instant our call for united action is described as an attempt "to sow dissension in our ranks" and "the use of the influence of The Hospital newspaper to upset the principle of the registration of individual nurses" and "to prevent nurses having this measure of justice." Such direct misrepresentation is unworthy of the editor of any journal which claims to speak on behalf of British nurses, who will naturally refuse to follow leaders who pursue tactics of this description. Abuse is not argument, and unless those responsible for the mis-statements we have quoted discontinue the practices of what are known in America as "the yellow journals" they must fail to exercise any influence for good in nursing affairs in this country. We have never failed to do justice to all parties in the discussion on nursing affairs which has been proceeding with increasing vehemence during many years, and we hope, in view of the difficulties to be solved, that all parties may henceforward show good temper and courtesy, however much they may differ as to the policy which will best promote the well-being and prosperity of all classes of nurses. It will need all the influence of the wisest and most knowledgable amongst us to arrive at the best solution of the many difficulties which surround the question of nurse registration in this country at the present time.

### English Nurses for English Colonies.

Before Parliament was prorogued last Friday an issue of some importance was raised by an Ulster member who asked the Colonial Secretary whether the High Commissioner of Southern Nigeria acted upon his own initiative in inviting at the beginning of the present year a sisterhood of French nurses to Calabar to take charge of the Native Government Hospital, where the inmates are not Roman Catholics; for what period the nuns had been engaged; and whether it was intended to retain their services in preference to those of nurses who were not connected with religious orders. Mr. Lyttelton having explained that the proposal to employ two Roman Catholics as nurses in the Native Hospital at Calabar, was originally made to the Protectorate Government by the head of the Roman Catholic Mission two years ago, said that previously there had been no women nurses in the institution, and, as the proposal received the support of the principal medical officer and of the Acting High Commissioner, it was accepted on terms approved by him. The Colonial Secretary said that no undertaking was given in the terms of agreement that the sisters would be employed for a stated period, but he added that if the system works well he sees no reason why it should be changed. We quite understand the difficulty of making a change in the circumstances described, but with reference to the employment of nurses in Government hospitals for which this country is responsible, we certainly think that foreigners should not be selected for the work unless or until it is found that the services of fully-qualified Englishwomen cannot be obtained. This is a question which merits the attention of the Colonial Nursing Association.

### The Nurses' Bookshelf.

Outlines of Routine in District Nursing. By Miss M. Loane, Superintendent of District Nurses, Portsmouth. (London: The Scientific Press, Limited, 28 Southampton Street, Strand. Price 1s. 6d. net.)

This book has been written for the use of district probationers and private nurses. Miss Loane has treated her subject in a novel and admirably practical manner. It would almost seem as if she had consciously or unconsciously adopted the model of a cookery book for the practical teaching of nursing details, so concise and categorical is the arrangement of the lessons. The information throughout is arranged uniformly. Each chapter contains: First, a list of appliances, then follows a description of the method of application, and lastly come notes which draw attention to the points to be observed in the circumstances under consideration. In practice nothing could be more useful to probationer or nurse than the information and the method in which it is conveyed. The sixty-eight short lessons contain instruction upon almost every duty which a nurse finds herself called to perform. It is, of course, not intended in any way to supersede the text-books on nursing which are essential to complete a nurse's education. Miss Loane's book is exceedingly valuable as a supplementary work of ready reference to be used during the practice of a nurse's calling. It is generally admitted that institutional experience and teaching does not fit the nurse to meet the difficulties of private and especially district work. Miss Loane's great experience as a district nurse and teacher of district nurses has enabled her to recognise the difficulties that have to be encountered, and in her book she has done much to anticipate and remove these difficulties, and so complete is the information she gives that every nurse might with advantage possess Miss Loane's book. There may be a few points which afford an opportunity for difference of opinion in the procedure recommended, but the general teaching and recommendations are so admirable that the value of the work is unaffected. No teacher is infallible, and every nurse finds herself called to modify some of the lessons she has learnt. The book concludes with an Appendix devoted to the conduct, organisation, and appliances relating to district work. In this Appendix Miss Loane abandons the categorical form of the lessons and gives some admirable advice and suggestions. Her ideals are high; but, if all cannot attain perfection, the standard of nursing cannot be too high, and precept is both valuable and acceptacle from one who, like Miss Loane, possesses sympathy, interest, and experience in the work to which she has devoted her life.

### Everybody's Opinion.

[Correspondence on all subjects is invited, but we cannot in any way be responsible for the opinions expressed by our correspondents. No communication can be entertained if the name and address of the correspondent are not given as a guarantee of good faith, but not necessarily for publication. All correspondents should write on one side of the paper only.]

### LADY DOCTORS AND MIDWIVES.

"C. M. B." writes: Having read "J. W.'s" letter on lady doctors attending midwifery cases for 5s., I wish to say that I endorse all she has stated on the subject. I am a widow with a child to support, and took up midwifery seven years ago, having paid a large fee and gone through the usual hospital training, as a means of livelihood. I think it is very unfair for lady doctors to try and take the only support from widows and other women who are obliged by circumstances to work hard for a living, and all nurses who follow this profession will agree with me that the work is hard and badly paid without having the fees cut down. All fair-minded people should set their faces against this as a crying injustice to a large body of deserving and struggling women.

### NURSING IN A MILITARY HOSPITAL.

"A Kentish District Nurse" writes: I was called to a soldier at a Good Templar's fête on Monday last. The messenger said he was suffering from an attack of neuralgia. On my arrival he had recovered considerably. I was given to understand that several persons held him down, as he struggled violently. He had had a similar fit the night previous and his comrade had been up with him most of the time. He had a very rapid pulse, but the beats were not distinct, and his temperature was 99·2. I advised him to go to the hospital on his return home, but he would not do so because he said that if he did he would have to get up and do a certain portion of the work in the hospital as well as his bed, etc., and that patients suffering from pneumonia (temperature 105°) had to do the same. He added that the orderlies would not do these things for such patients; it had to be done and patients back to bed before the doctor's arrival; several of his comrades corroborated this statement. The wives of some of the soldiers complained also of ill-attention when ill, or in midwifery cases, where a midwife called could not manage. I should like to know if this is general in military hospitals.

### THE AVERAGE COST OF DIETS.

The Matron of the Isolation Hospital, Foxby Hill, writes: Having read the account of the daily average for food at the Folkestone Sanatorium, may I state I consider it heavy? As matron for some years both in fever hospitals and also in convalescent homes I have found that by having a conscientious woman in the kitchen one can feed staff and patients well on 8½d. to 9½d. per head daily, Christmas month coming out at 10d. on account of little extras. It is false economy having young and inexperienced servants, as the waste they entail is enormous. I pay full price for everything, as we do not have any of the food by contract, and as far as I am able I buy the best of everything, and of course vary it as much as possible. I am fortunate in having a splendid committee, who leave the management of the housekeeping, etc., to me, and I and my staff work happily together; but then I am not put on a level with the gardener, as the Corporation of Folkestone seems to think a matron should be. I consider all matrons should be gentlewomen and fully trained, and I think if they are treated with respect and consideration by their committee—like I am—they in turn will study them, and do their utmost to manage the hospital efficiently.

### Appointments.

[No charge is made for announcements under this head, and we are always glad to receive and publish appointments. The information, to insure accuracy, should be sent from the nurses themselves, and we cannot undertake to correct official announcements which may happen to be inaccurate. It is essential that in all cases the school of training should be given.]

Isolation Hospital, Basingstoke. — Miss Annie Jenkins has been appointed nurse matron. She was trained under the Metropolitan Asylums Board and at Ancoats and Aldwick Hospital and Dispensary, Manchester. She has since been matron of the Tours Sanatoria, Guernsey, for enteric and diphtheria patients, and charge nurse at the Rhondda Fever Hospital, South Wales.

Isolation Hospital, Chadwell.—Miss Barding has been appointed matron of the above institution. She was trained at St. Mary's Hospital, Paddington, and acted subsequently as sister at the Victoria Hospital, Folkestone, and as matron at the Borough Sanatorium, Folkestone.

Isolation Hospital, Ilford.—Miss C. Alice Barling has been appointed matron. She was trained at St. Mary's Hospital, Paddington, and was afterwards temporary theatre sister. She has since been sister at the Victoria Hospital, Folkestone, and matron of the Borough Sanatorium, Folkestone.

Isolation Hospital, Rothwell, near Leeds.—Miss Jennie Earley has been appointed sister. She was trained at the City Hospital, Nottingham, and has since been charge nurse at the Small-pox Hospital, Bulwell Forest, Nottingham, and charge nurse at the Isolation Hospital, Rothwell, Leeds.

Malton Cottage Hospital.—Miss Lilian Lloyd has been appointed matron. She was trained at the Jessop Hospital for Women, Sheffield, and the General Infirmary, Bolton, Lancashire, and has since been charge nurse at the Park Fever Hospital, London; night superintendent at the Royal Infirmary, Halifax; and night superintendent at the Royal Hospital for Diseases of the Chest, London.

Newton Abbot Isolation Hospital—Miss M. Elliott has been appointed nurse-matron. She was trained at the Richmond, Whitworth, and Hardwicke Hospitals, Dublin, and has since been sister at the Birmingham Infirmary, sister at Bucknall Fever Hospital, and superintendent nurse at Cheadle Workhouse Infirmary. She has also done private nursing.

Royal National Hospital for Consumption, Ireland.—Miss Margaret Brown has been appointed night superintendent. She was trained at Forfar Infirmary and Dundee Royal Infirmary, where lately she did sisters' holiday duty. She has also been sister at Ruchill Fever Hospital, Glasgow.

Royal National Hospital for Consumptives, Ventnor.—Miss Elizabeth Davies has been appointed matron. She was trained at Chelsea Infirmary, where she has since been assistant matron. She has also been matron at Essex and Colchester Hospital, Colchester.

Royal Victoria Hospital, Dover.—Miss G. M. Cubitt has been appointed sister. She was trained at Coventry and Warwickshire Hospital, and has since been staff nurse at the Royal Halifax Infirmary, charge nurse at the Oxford Eye Hospital, head day nurse at Worthing Hospital, where she has also taken matron's holiday duty.

Thomas Walker Hospital, Fraserburgh, N.B.—Miss Jessie Taylor has been appointed matron. She was trained at the Royal Infirmary, Aberdeen, and afterwards worked on the private staff of the Northern Nursing Home at Aberdeen for five years.

Workhouse Infirmary, Ipswich.—Miss Martha Mawson has been appointed charge nurse. She was trained by the Northern Nursing Association, and has since been charge nurse at the Williton Workhouse Infirmary, Somersetshire.

Worthing Isolation Hospital.—Miss R. A. Hall has been appointed nurse-matron. She was trained at the Cancer Hospital, Brompton, and London Hospital, Whitechapel, and was afterwards attached to the private staff of the latter. She has since been sister-in-charge and night superintendent at the Plaistow Isolation Hospital, and has also taken assistant matron's holiday duty.

### Presentations.

Derbyshire Royal Infirmary, Derby.—Miss Florence Keene having resigned her post as sister of the male medical ward in Derbyshire Royal Infirmary to take up her post as matron of the Penistone District Isolation Hospital, Sheffield, was presented by the matron, the nursing staff, house physician, and secretary-superintendent with a royal crown Derby tea service and tray, copper kettle and stand, card tray, and silver sugar tongs; a travelling clock, royal crown Derby tea pot and preserve dish by the honorary physicians, and many other valuable and useful presents given by her special friends.

### TRAVEL NOTES AND QUERIES.

By our Travel Correspondent.

Paying Guest in France (Chris).—I can give you help in what you want, but must first know much more as to your requirements. Write me fully, first, how much can you afford to pay per week; second, what are your tastes—that is, do you prefer town or country life, and would you, on the whole, like Switzerland better than France. If you wish to learn French rapidly, it would be better to be where no English is spoken.

The Canary Islands (Ishtal).—With regard to clothing you will want chiefly light woollen garments. At that season it is not hot, but is something like a fine October with us. Certainly muslins will not be required. Well-made thin woollen dresses of various thicknesses are what you need. As to hotels I will tell you later on. I am now awaiting answers to inquiries I have made on this subject.

Jersey for a Week (Matron).—You do not repeat your needs, but if my memory serves me correctly the intending travellers are three nurses with a brother. If they can only afford the sum you mention I think they are making a mistake in trying to see Guernsey as well as Jersey. The Eastern and Western Railways in Jersey enable you to see the country very well and coach drives complete the circuit of the island; it is so small that three drives embrace all the most important points. In Guernsey Victor Hugo's house called "Hauteville" is one of the lions. From St. Peter-Port there is an electric tram to St. Sampson's Harbour, about four miles distant, and there are car drives also which encircle the island. Messrs. A. and C. Black publish a useful little guide book, price 1s., to the Channel Isles, which your travellers should get; it will give them all necessary help.

Rules in Regard to Correspondence for this Section.—All questioners must use a pseudonym for publication, but the communication must also bear the writer's own name and address as well, which will be regarded as confidential. All such communications to be addressed "Travel Correspondent, 28 Southampton Street, Strand." No charge will be made for inserting and answering questions in the inquiry column, and all will be answered in rotation as space permits. If an answer by letter is required, a stamped and addressed envelope must be enclosed, together with 2s. 6d., which fee will be devoted to the objects of "The Hospital" Convalescent Fund. Ten days must be allowed before an answer can be published.

### Notes and Queries.

**REGULATIONS.**

**The Editor is always willing to answer in this column, without any fee, all reasonable questions, as soon as possible.**

**But the following rules must be carefully observed.**

**1. Every communication must be accompanied by the name and address of the writer.**

**2. The question must always bear upon nursing, directly or indirectly.**

**If an answer is required by letter a fee of half-a-crown must be enclosed with the note containing the inquiry.**

*Pelvis and Fœtal Skull.*

(160) Could I trouble you for the address of a place where I could hire a pelvis and fœtal skull on reasonable terms?—*L. S.*

Write to Messrs. S. Maw, Son, and Sons, 7-12 Aldersgate Street, London, E.C.

*Holt-Ockley System: Medicines.*

(161) 1. Will you kindly explain the meaning of "Holt-Ockley" system? 2. Can you tell me the name of any book on poisons and their antidotes, and the technical names of medicines?—*Peter.*

1. Write to the Secretary of the Holt-Ockley Nursing Association, 12 Buckingham Palace Road, S.W. 2. Dock's "Materia Medica for Nurses," published by Putnam, and obtainable from the Scientific Press. Price 3s. 6d. net.

*Infirmaries.*

(162) Will you kindly tell me whether all the infirmaries mentioned in your book "How to Become a Nurse" are necessarily Poor-law ones? Also whether the training at such infirmaries is considered to give one such a good standing afterwards as that of a general hospital, and could one go as nurse or sister to a general hospital after training as a probationer at an infirmary?—*G. C.*

The infirmaries mentioned are not all Poor-law institutions. In the provinces many general hospitals are called infirmaries, and the training given at such ranks high. Nurses who have been trained in Poor-law infirmaries often obtain posts in general hospitals.

*Guild of St. Barnabas.*

(163) I shall be much obliged if you can give me any particulars about the Guild of St. Barnabas for Nurses, or where I can apply for information as to how to become a member.—*Faith.*

This Guild enrols trained nurses and midwives who are members of the Church of England. There are branches in India, England, and the Colonies. For particulars, write to the Hon. Secretary, Nurses' Hostel, Francis Street, London, W.C.

*Finsen Light Treatment.*

(164) Will you kindly tell me in which hospital the Finsen Light treatment for lupus is given? I have a child of eight in my district who is suffering from it?—*Nurse S. A. F.*

At Guy's Hospital, the London Hospital, and others.

*Convalescent Homes.*

(165) Will you kindly give me the addresses of some convalescent homes on the south and east coast. I am a district nurse and have some difficulty in finding suitable places for my convalescent patients.—*Lucy.*

"Burdett's Hospitals and Charities," published by the Scientific Press, Limited, contains a full list of such homes.

*Midwifery.*

(166) Will you kindly tell me where I could be trained in midwifery, not by the Ockley system, nor by paying a premium?—*S. W.*

Write to the Secretary, Rural Midwives Association, 47 Victoria Street, London, S.W.

*Surgical Work.*

(167) Can you tell me of any hospital or institution where I can get three or six months' good all-round experience in surgical work only? I have had three years' training in medical and maternity work, but now require surgical training. I could not afford to pay a fee, but would give services.—*Nurse M.*

Write to the Matron, Bolingbroke Hospital, Wandsworth Common, S.W.

**Handbooks for Nurses.**

| | Post Free. |
|---|---|
| "A Handbook for Nurses." (Dr. J. K. Watson.) ... | 5s. 4d. |
| "Nurses' Pronouncing Dictionary of Medical Terms." ... | 2s. 0d. |
| "Art of Massage." (Creighton Hale.) ... ... ... | 6s. 0d. |
| "Surgical Bandaging and Dressings." (Johnson Smith.) | 2s. 0d. |
| "Hints on Tropical Fevers." (Sister Pollard.) ... ... | 1s. 8d. |

Of all booksellers or of The Scientific Press, Limited, 28 & 29 Southampton Street, Strand, London, W.C.

### For Reading to the Sick.

"IN ALL TIME OF TRIBULATION."

Father, my cup is full,
My trembling soul I raise;
Oh, save me in this solemn hour,
Thy might and love to praise!

Father, my cup is full,
But thou dost bid me drink;
I know Thy love the chalice mixed,
And yet I faint—I shrink.

Father, forsake me not!
O Christ! I look to Thee;
And by Thy midnight agony
Do Thou remember me.

*A. Shipton.*

Though the history of our divine Lord's life on earth is chiefly taken up with the details of His public ministry—though He is most often presented to our view as the great Teacher and Healer of man, while His own inner and individual human existence (if we may so speak) seems hidden from our gaze—yet, ever and anon, as we meditate more and more on the holy Gospels, the veil is lifted which shrouds the mystery of His daily life, and there shine out from beneath it those tender sympathies, those touching incidents and lowly details of a human and suffering life, which "draw us with the cords of a man, with the bands of love," to Him who has borne our nature even to the heavenly throne.

We know that He lays on us no burden which He has not borne before; He does not even tell us, as earthly friends would sometimes do, that pain is not wearisome, and that sorrow is not sad; ah no! He knows their bitterness too well for that. In the storehouse of His loving heart he treasures still the memories of earth, of His sorrows and of His pains; they have lost now their sharpness and their sting; the crown of thorns has budded (as Aaron's rod of old) into tender blossoms of everlasting light; but He bears in His hands and in His side the tokens of His love unto death, the marks of "the wounds wherewith He was wounded in the house of His friends.

He only asks that what He bore for us we would bear in our turn for Him (a light cross, indeed, compared with His heavy one, and yet He knows that we could bear no more)—He only asks us to rest on Him when our hearts are failing us for fear; only to remember Him in His agony, and never to forget Him in our joy; to be partakers of His sufferings and His sympathy here, till the burden of our cross shall be changed into the "exceeding and eternal weight of glory," which we shall bear, by the help of His love, for ever in heaven.

*M. E. S.*

When the springs of life are worn,
When the waves are sighing,
That the night is almost gone,
For the lights are dying.
Standing on the shore at dawn
Of the Resurrection Morn
With Thyself, all need supplying,
Bid me "Come,"
Lord Jesu!

*Anon.*

## Figures and Tables

**7. f1:**
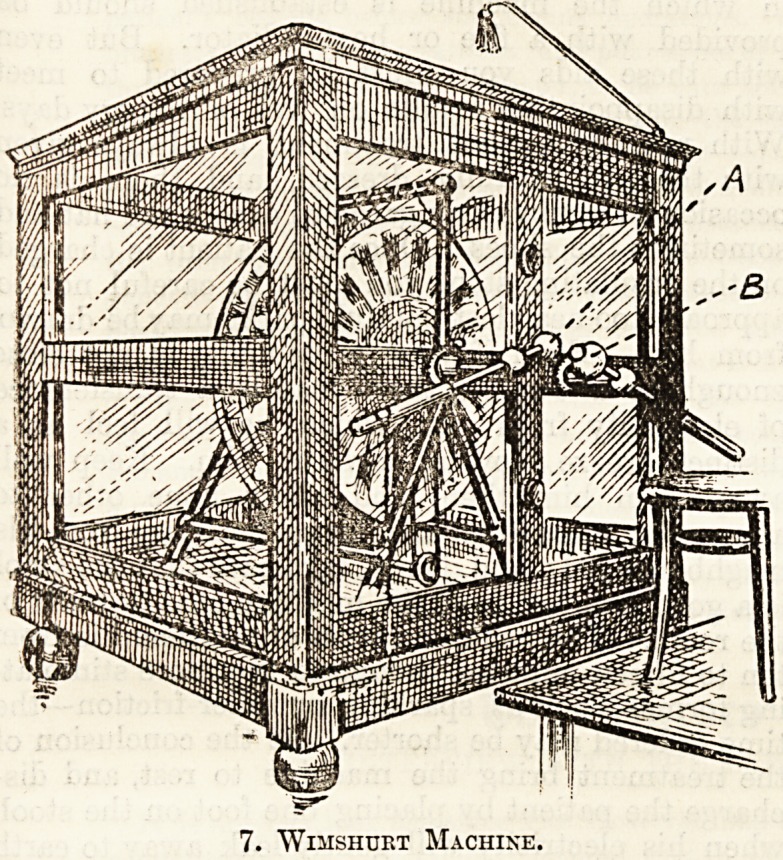


**8. f2:**
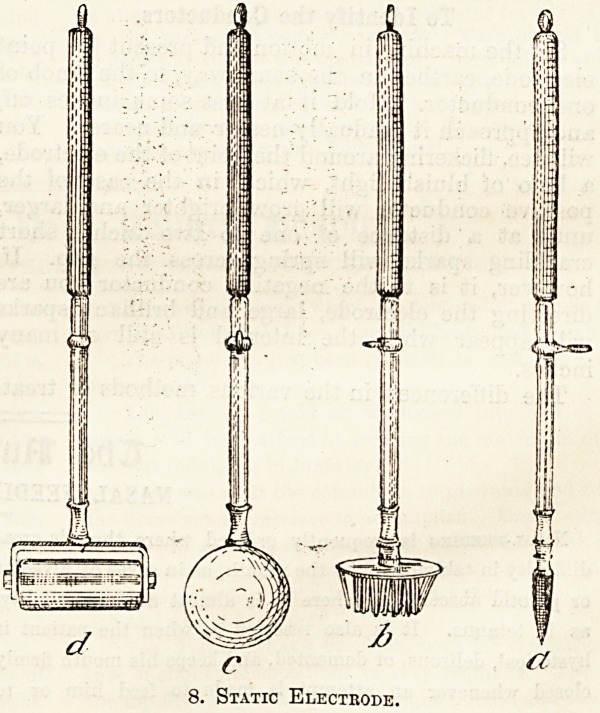


**Figure f3:**
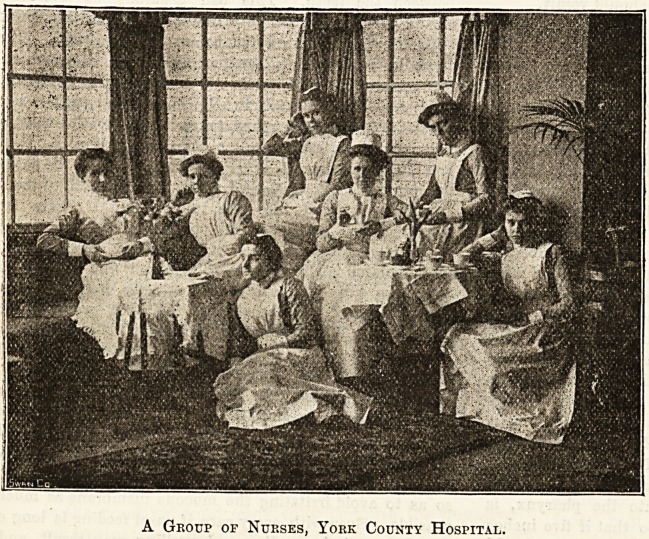


**Figure f4:**